# Identification of a novel iron regulated basic helix-loop-helix protein involved in Fe homeostasis in *Oryza sativa*

**DOI:** 10.1186/1471-2229-10-166

**Published:** 2010-08-11

**Authors:** Luqing Zheng,, Yinghui Ying, Lu Wang, Fang Wang, James Whelan, Huixia Shou

**Affiliations:** 1 Joint Research Laboratory in Genomics and Nutriomics, College of Life Sciences, Zhejiang University, Hangzhou 310058, P R China; 2 State Key Laboratory of Plant Physiology and Biochemistry, College of Life Sciences, Zhejiang University, Hangzhou, 310058, P R China; 3 Australian Research Council Centre of Excellence in Plant Energy Biology, University of Western Australia, Crawley 6009, WA, Australia

## Abstract

**Background:**

Iron (Fe) is the most limiting micronutrient element for crop production in alkaline soils. A number of transcription factors involved in regulating Fe uptake from soil and transport in plants have been identified. Analysis of transcriptome data from *Oryza sativa *grown under limiting Fe conditions reveals that transcript abundances of several genes encoding transcription factors are altered by Fe availability. These transcription factors are putative regulators of Fe deficiency responses.

**Results:**

Transcript abundance of one nuclear located basic helix-loop-helix family transcription factor, *OsIRO3*, is up-regulated from 25- to 90-fold under Fe deficiency in both root and shoot respectively. The expression of *OsIRO3 *is specifically induced by Fe deficiency, and not by other micronutrient deficiencies. Transgenic rice plants over-expressing *OsIRO3 *were hypersensitive to Fe deficiency, indicating that the Fe deficiency response was compromised. Furthermore, the Fe concentration in shoots of transgenic rice plants over-expressing *OsIRO3 *was less than that in wild-type plants. Analysis of the transcript abundances of genes normally induced by Fe deficiency in *OsIRO3 *over-expressing plants indicated their induction was markedly suppressed.

**Conclusion:**

A novel Fe regulated bHLH transcription factor (OsIRO3) that plays an important role for Fe homeostasis in rice was identified. The inhibitory effect of *OsIRO3 *over-expression on Fe deficiency response gene expression combined with hypersensitivity of *OsIRO3 *over-expression lines to low Fe suggest that OsIRO3 is a negative regulator of the Fe deficiency response in rice.

## Background

Iron (Fe) is an essential micronutrient for plant growth and production. This is due to the fact that it is an essential co-factor in a variety of enzymes that play critical roles in photosynthesis, respiration and nitrogen fixation [1]. Although Fe is the second most abundant metal element in the earth crust, its bio-availability is limited, especially in alkaline soils where Fe largely exists as insoluble hydroxides or oxides [2]. While the optimal Fe concentration for plant growth is in the range of 10^-9 ^to 10^-4^M , the bio-available Fe in most soils is approximately 10^-17 ^M [2,3]. Plants have two distinct uptake strategies to increase the efficiency of Fe uptake from soil [4]. The reduction strategy employed by non-grass plant species uses a Fe deficiency induced reductase to convert insoluble Fe(III) to Fe(II), the latter being transported into plant cells by the Fe(II) transporter IRT1 [4-6]. In contrast, grass species use a chelating strategy to obtain Fe from soil. The chelating strategy consists of Fe deficiency induced biosynthesis and secretion of phytosiderophore(s) and cognate high affinity transporters, Fe(III)-phytosiderophore Yellow Stripe Transporter, ZmYS1, HvYS1 and OsYSL15 [7-9].

Many of the components involved in Fe uptake for both of the strategies outlined above have been identified at a molecular level. In Arabidopsis H^+^-ATPase 2 (AHA2) that mediates acidification of the rhizosphere [10], a ferric reductase FRO2 [6] and a ferrous Fe transporter IRT1 [5], represent the three key components required for a strategy I Fe uptake system. Identification of the molecular components involved in strategy II Fe uptake system has focused on the biosynthesis of the Fe(III)-chelator, phytosiderophore (PS) [11]. Key genes in the PS biosynthesis include nicotianamine synthase gene *OsNAS1/2/3 *[12], nicotianamine aminotransferase gene *OsNAAT1 *[13], and the cognate transporter Yellow-Stripe 1 [7].

Many of the genes involved in Fe uptake via strategy I or II display a distinct Fe deficiency induced expression pattern [14]. Transcriptome studies have shown that large scale alterations of transcript abundances is a common feature of Fe deficiency and thus plays a key role in the Fe deficiency response [15-17]. Alterations in transcript abundances of genes encoding transcription factors are of particular interest in these studies due to their potential role in regulating the Fe deficiency response. The bHLH transcription factor FER, a regulator of iron uptake, was first identified from the analysis of the tomato *fer *mutant [18]. The *fer *mutant failed to activate a Strategy I Fe uptake pathway under Fe deficient conditions. Studies have also shown that either FER or its Arabidopsis ortholog, FER-like transcription factor, FIT1, is required for the strategy I responses [19]. Subsequent studies have identified a family of bHLH transcription factors in Arabidopsis (AtbHLH 38/39/100/101) that interact with FIT1 [20,21]. A number of transcription factors involved in regulating the Fe deficiency response in *Oryza sativa *(rice) have also been identified. The Fe-regulated bHLH transcription factor, OsIRO2, shares high similarity with the Arabidopsis family of bHLH transcription factors and positively regulates the expression of strategy II pathway genes, including *OsNAS1/2/3*, *OsNAAT1*, deoxymugineic acid synthase gene *OsDMAS1 *and a YS-like gene *OsYSL15 *[22]. Two other transcription factors, Fe-deficiency-responsive factors, IDEF1 and IDEF2, bind the Fe-deficiency-responsive elements 1 and 2 (IDE1 and IDE2) and positively regulate a large number of Fe responsive genes [23-25]. As *IDEF1 *and *IDEF2 *are constitutively expressed in roots and shoots in rice, other regulatory factors which may only be expressed under Fe limiting conditions may be involved in regulating the response to Fe deficiency.

A previous study identified transcripts of several genes encoding transcription factors that changed in abundance with Fe deficiency [17]. In this study, the function of one of the transcription factors, a bHLH transcription factor (TF) family protein, named OsIRO3 (OsIRbHLH2 in previous paper, LOC_Os03g26210) was investigated. Our analysis suggests that OsIRO3 acts as a negative regulator of the Fe deficiency response.

## Results

### *OsIRO3 *is specifically induced by Fe deficiency

**Figure 1:**
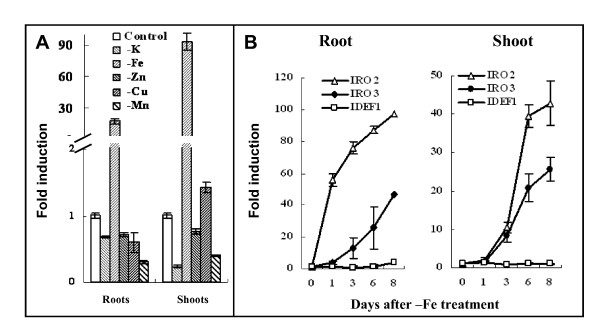
**Expression analysis of *OsIRO3***. Three week old rice seedlings were transferred to solution culture with different metal element deprivation (-K, -Fe, -Zn, -Cu, -Mn) for eight days. Root and leaf samples were separately analyzed and the control expression level was normalized to 1 in both root and leaves. (A) Expression of *OsIRO3 *under different nutrient deficiency conditions. (B) Time-course transcript abundance of *OsIRO3*, *OsIRO2 *and *IDEF1 *in response to Fe deficiency.

Previous studies in our laboratory identified several transcription factors whose expression was induced by Fe deficiency in rice [17]. One gene encoding a bHLH transcription factor, *OsIRO3*, is highly induced by Fe deficiency in both roots and shoots [17]. To determine whether the induction of *OsIRO3 *is specific to Fe deficiency or whether this induction could be observed under deficiency of other minerals, the transcript abundance of *OsIRO3 *was determined from plants grown under a variety of different mineral element deficient conditions. The transcript abundance of *OsIRO3 *was markedly induced by Fe deficiency in both roots and shoots, by 25- and 90-fold respectively (Figure 1A). Deficiency of several other minerals, including copper and zinc had no effect of the induction of *OsIRO3 *in roots or shoots (Figure 1A), indicating that the induction of *OsIRO3 *was specific to Fe deficient conditions.

The time-course expression pattern of the *OsIRO3 *and the other two known transcription factors, *OsIRO2 *and *IDEF1*, in response to Fe deficiency was investigated. Consistent with previous studies [15,22,24], *IDEF1 *was constitutively expressed in both roots and shoots (Figure 1B). In contrast *OsIRO2 *and *OsIRO3 *were significantly up-regulated by Fe deficiency, with a 10-fold induction or greater evident after 3 days and continued induction up to 8 days (Figure 1B). The induction of *OsIRO3 *was less in terms of magnitude in shoots, *OsIRO3 *was induced 20-fold compared to *OsIRO2 *being induced almost 40-fold after 6 days (Figure 1B shoot). In roots *OsIRO3 *induction lagged behind that of *OsIRO2 *both in time and magnitude, *OsIRO2 *was already induced 50-fold after 1 day compared to *OsIRO3 *for which a 10-fold induction was observed only after 3 days (Figure 1B root).

### Subcellular localization of *OsIRO3*

**Figure 2:**
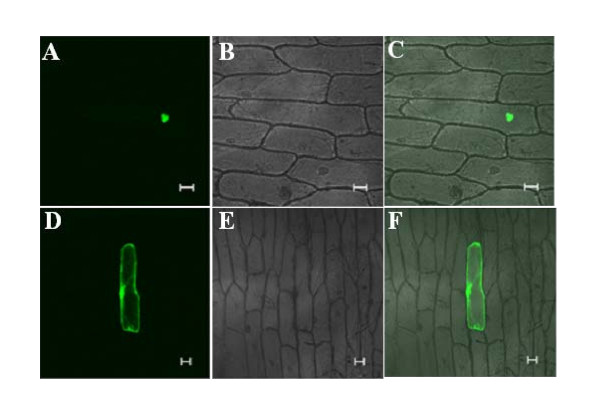
**Subcellular localization of OsIRO3**. Confocal images of onion epidermis cells expressing *OsIRO3-sGFP *(A, B, C) and *sGFP *(D, E, F). Images were taken under GFP channel (A and D), under transmitted light (B and E), and their merged images (C and F). Bar = 50 μm.

To determine the subcellular localization of OsIRO3 protein, the full length cDNA of *OsIRO3 *was cloned in frame to the 3' end of the *sGFP*. The expression of the fusion protein was driven by the 35S CaMV promoter. Targeting ability was tested in onion epidermal cells using microprojectile bombardment along with the vector control which contains *sGFP *alone. Results showed that the OsIRO3::sGFP fusion protein was targeted to nuclei (Figure 2A to 2C). The control sGFP was found throughout the cell, in nuclei and the cytosol (Figure 2D to 2F), indicating that OsIRO3 has specific nuclear targeting ability.

### Phylogenetic analysis of *OsIRO3*

**Figure 3:**
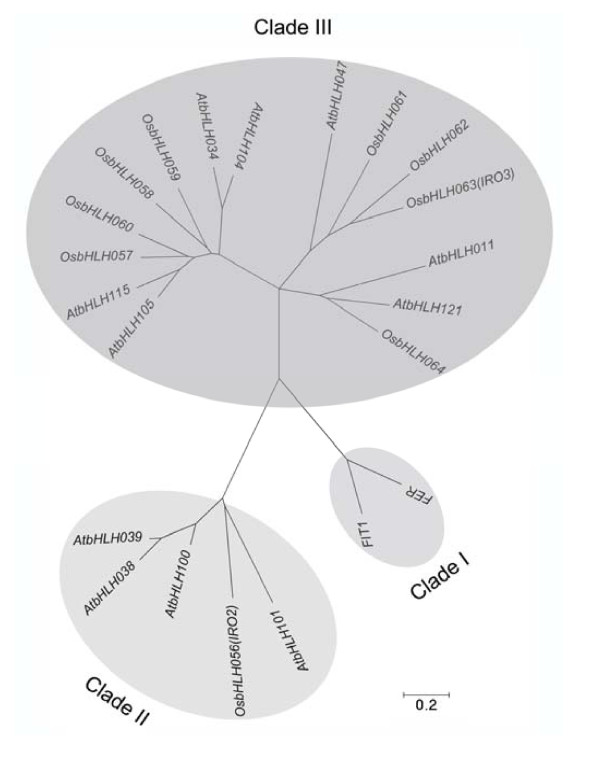
**Phylogenetic tree of OsIRO3-like bHLHs with known bHLH transcription factors that function in the Fe deficient response**. bHLH transcription factors from Arabidopsis and rice were subjected to the neighbor-joining algorithm phylogenetic tree construction using MEGA software version 4.1 [37] with default settings. The amino acid sequences used to generate this phylogenetic analysis are listed in Additional file 1.

There are 158 and 173 predicted members of the bHLH-domain containing transcription factors in Arabidopsis and rice respectively [26,27]. To investigate the relationship between *OsIRO3 *and previously characterized bHLH transcription factors regulating the Fe deficient response in Arabidopsis and rice, a phylogenetic analysis using amino acid sequences of OsIRO3 and the previously characterized bHLH transcription factors tomato FER [18], Arabidopsis FIT1 [19], AtbHLH 38, 39, 100 and 101 [20] was performed (Amino acid sequences were listed in Additional file 1). As expected, FER and its Arabidopsis ortholog, FIT1 were classified into the same Clade (Figure 3, Clade I). OsIRO2 branches in the same clade with the Arabidopsis bHLH family proteins 38/39/100/101 (Figure 3, Clade II). OsIRO3 branches into Clade III (Figure 3). AtbHLH105 (ILR3) which was reported to modulate metal homeostasis and response to auxin was also found in this clade [28]. The protein sequence of Arabidopsis ILR3 is only 20.9% identical to OsIRO3. Also, OsbHLH62 (OsbHLH1), that branches closest to OsIRO3 (Figure 3), has been previously reported to be induced by cold stress [29].

### Over-expressing *OsIRO3 *results in hypersensitivity to Fe deficiency

**Figure 4:**
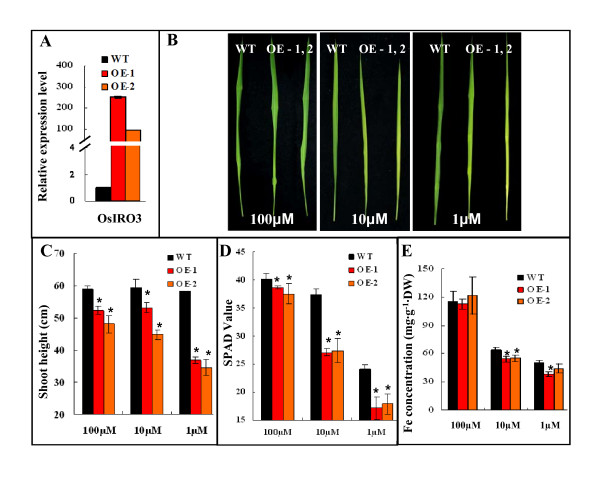
**Growth performance and transcript abundance of *OsIRO3 *in over-expressing (OE) lines**. Three week old transgenic and vector only transformed rice plants, the latter referred to as wild-type, were transferred to solution culture containing 100, 10, or 1 μM EDTA-Fe for 10 days. (A) Expression levels of *OsIRO3 *in OE plants grown in normal solution culture compared to wild-type. The expression level of wild-type was normalized to one. (B) The youngest leaf of *OsIRO3*-OE and wild-type plants in solution culture with 100 μM, 10 μM, or 1 μM Fe-EDTA. (C) Shoot height of *OsIRO3-*OE and wild-type under the various Fe conditions. (D) SPAD values of newly expanded leaves of *OsIRO3*-OE and wild-type plants. (E) Shoot Fe concentrations of *OsIRO3*-OE lines and wild-type.

*OsIRO3 *was over-expressed (OE) in rice to investigate the role it played in Fe homeostasis. Two independent OE transgenic lines with significantly altered expression level of *OsIRO3 *(Figure 4A) were studied in detail. To examine if alteration of *OsIRO3 *expression in transgenic lines influences tolerance to low Fe conditions, three week old OE and wild-type rice seedlings were transferred into solution culture containing high (100 μM ), moderate (10 μM), or low (1 μM) levels of Fe for 10 days. Under Fe sufficient conditions (100 μM), the transgenic seedlings overexpressing *OsIRO3 *had significantly shorter shoots compared to the wild-type counterparts(Figure 4C, Additional file 2), although the color was normal with no evidence of chlorosis that would suggest Fe deficiency (Figure 4B, Additional file 2). SPAD values and Fe concentrations from these plants also confirm values very close to wild type, although SPAD values were slightly but significantly lower (Figure 4D and 4E). Under moderate Fe supply (10 μM), the two OE lines displayed a chlorotic phenotype in the newly developed leaves compared to the wild-type plants (Figure 4B, Additional file 2). The SPAD values of the youngest leaves in both OE lines under 10 μM Fe were reduced to ~ 65% compared to the wild-type control (Figure 4D). At 1 μM Fe supply, all plants displayed chlorotic symptoms (Figure 4B, Additional file 2). Both OE lines had significantly reduced SPAD values and shoot height compared to wild-type (Figure 4C and 4D, Additional file 2). The Fe concentration of shoots in both OE lines was significantly reduced compared to wild-type under both medium and low levels of Fe supply (Figure 4E).

### *OsIRO3 *suppresses the induction of Fe responsive genes by Fe deficiency

**Figure 5:**
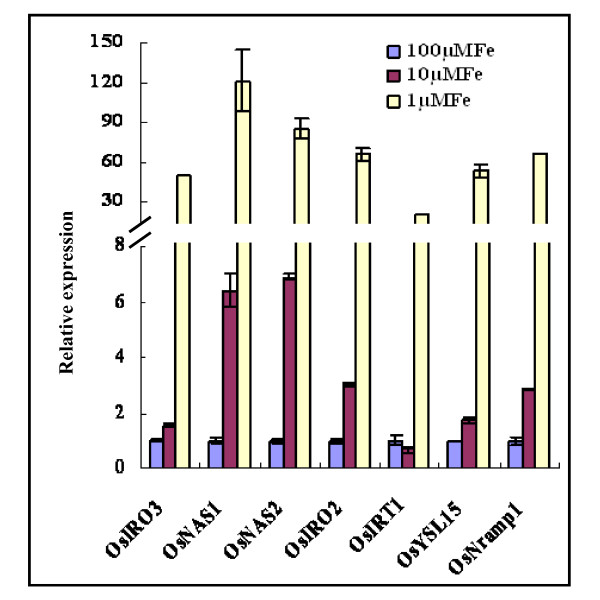
**Expression patterns of Fe deficiency response genes in wild type roots grown under different Fe concentrations**. Three week old wild-type rice plants were subjected to solution culture containing 100, 10, or 1 μM EDTA-Fe for 10 days. Root RNA was prepared for quantitative RT-PCR analysis. For each gene transcript abundance measured under 100 μM EDTA-Fe was normalized to one with other values shown as relative expression.

**Figure 6:**
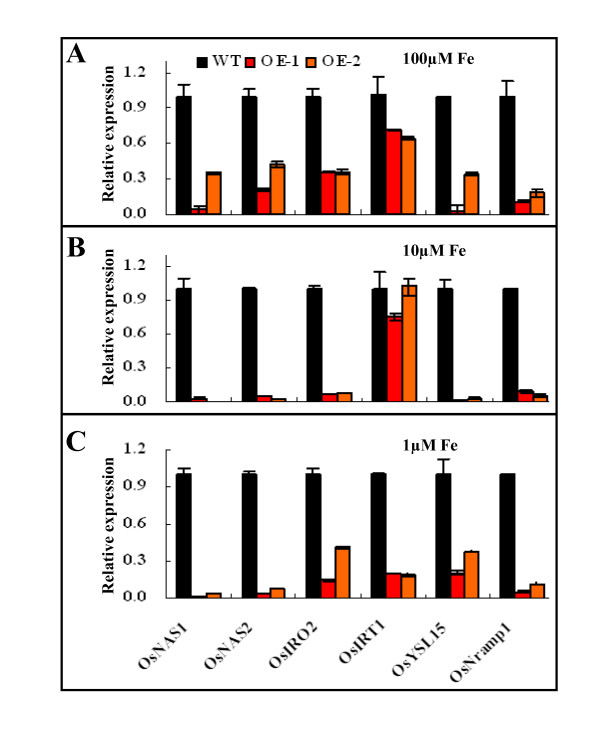
**Expression patterns of Fe deficiency response genes in roots of *OsIRO3*-OE and wild-type plants under 100 μM (A), 10 μM (B), or 1 μM Fe-EDTA (C) conditions**. Plant growth conditions are described in detail in material and method. For each gene transcript abundance measured for wild type plants grown under 100 μM EDTA-Fe was normalized to one with other values shown as relative expression.

The expression patterns of previously characterized Fe deficiency responsive genes, *OsNAS1, OsNAS2, OsIRO2, OsIRT1, OsYSL15*, and *OsNramp1 *[5,6,8,12,30] were investigated under different Fe levels. As expected, all of these genes were markedly induced by Fe deficiency (Figure 5). *OsNAS1 *and *OsNAS2 *were very sensitive to Fe availability, displaying the highest induction levels under Fe deficiency conditions (Figure 5). However, in the two OE *OsIRO3 *transgenic lines, the induction of transcript abundance for these genes, with the exception of *OsIRT1 *in roots, was significantly suppressed under all three Fe supply conditions (Figure 6A-C). The expression of *OsIRT1 *was only significantly down-regulated by both OE lines under low Fe supply condition (Figure 6C).

## Discussion

Several lines of evidence indicate an important regulatory role for OsIRO3 in response to Fe deficiency in rice. Firstly, it is specifically induced by Fe deficiency but not by other metal depletion. Secondly, *OsIRO3*-OE lines are more sensitive to low Fe availability than wild-type. Thirdly, the induction of Fe deficiency responsive genes that play pivotal roles in Fe uptake are suppressed in *OsIRO3*-OE lines. Together these data imply that OsIRO3 is a negative regulator of Fe uptake in rice.

To date, a variety of bHLH transcription factors have been shown to play important roles in regulating Fe uptake and homeostasis [15,18,20-22]. OsIRO3 is not phylogenetically related to other known bHLH transcription factors involved in the regulation of Fe uptake, such as *OsIRO2 *and *IDEF1 *[15,24]. Thus, it is a novel Fe-responsive transcription factor. Over-expression of *OsIRO2 *and *IDEF1 *increased the expression of Fe uptake related gene transcripts and these plants also showed an enhanced tolerance to Fe deficiency. Thus, they are positive regulators of downstream Fe responsive genes [24]. In contrast, this study revealed that over-expression of *OsIRO3 *results in hypersensitivity to Fe deficiency, due to the suppression of induction of Fe uptake related genes, suggesting it is a negative regulator. Fe deficiency induces the activation of the methionine cycle and biosynthesis of PS in strategy II plants [14,17], a process which is nutrient and energy demanding. Over-expression of NA synthase alone, an enzyme catalyzing a rate limiting step of NA and PS synthesis resulted in significantly reduced growth [31,32]. In addition, excessive Fe acts as a catalyst for the formation of free radicals that are potent oxidizing agents which can damage many important biological molecules in cells [2]. Thus, although the Fe deficiency response results in increased Fe uptake it also results in a growth penalty. Therefore negative regulators are required to balance the induction of Fe uptake components to reduce the negative effect on plant growth that results from excessive Fe and/or as a result of the induction of the components due to Fe deficiency. The fact that the induction of *OsIRO3 *is slightly later in shoots and at a lower level in roots compared to the positive regulator *OsIRO2 *(Figure 1B) suggests that OsIRO3 may play a role as part of a negative feed back loop on the Fe deficiency response.

The negative effect of OsIRO3 occurs via either a direct or indirect mechanism. As *OsIRO3 *induction lags that of *OsIRO2*, it may be regulated by OsIRO2 to form a negative feedback loop. In fact, there is a putative OsIRO2 binding *cis*-element in the promoter region of *OsIRO3 *(Additional file 3). bHLH transcription factors often function in homo- and/or hetero-dimerized forms [33,34]. OsIRO3 and OsIRO2 and/or IDEF1 regulate similar downstream targets, albeit in opposite manners. These transcription factors might interact with each other and/or bind to the same *cis*-acting regulatory elements in the promoter region of target genes. Analysis of the expression of *OsIRO3 *and *IDEF1 *in different microarray data sets showed that these two genes are expressed antagonistically (Additional file 4). There are a number of predicted proteins in Arabidopsis and rice with high sequence similarity to *OsIRO3 *(Figure 2). The closest orthologue of *OsIRO3 *gene in Arabidopsis, *AtbHLH047*, is also induced by Fe deficiency (unpublished data). Whether AtbHLH047 plays a similar role in Fe homeostasis in Arabidopsis needs to be investigated.

## Conclusion

This study reveals the regulatory role of a bHLH protein, OsIRO3 in Fe homeostasis in rice. *OsIRO3 *is specifically induced by Fe deficiency, suggesting it functions in Fe deficiency responses. The nuclear localization of OsIRO3 supports the hypothesis that OsIRO3 acts as a transcription factor. The hypersensitivity to low Fe supply of the *OsIRO3*-OE and the inhibitory effect of *OsIRO3*-OE on the induction of Fe deficiency response genes suggest OsIRO3 functions as a negative regulator in Fe deficiency responses. The identification of the new important regulator adds to a growing understanding of the network of fine control of Fe homeostasis.

## Methods

### Plant Materials

The rice cultivar Nipponbare was used in this study as a wild-type control. To construct a binary vector for the over-expression of *OsIRO3*, the full length cDNA sequence of the *OsIRO3 *gene was amplified using the following primers:

Forward 5'- GGATCCGATTTGAGCAGGGAACGGAAGG -3'

Reverse 5'- GGATCCGACAGAAGTGTTTTCGTGTGGA -3'.

The PCR fragment was then cloned into the Takara pMD19 vector. After sequence confirmation, it was cloned into pTF101-ubi at the *Bam*HI site (Additional file 5) [13]. Agrobacterium-mediated rice transformation of rice callus was carried out as described previously [35]. Ten independently transformed plant lines were generated. QRT-PCR analysis was performed to evaluate the effect of over-expression. Two independent lines were selected with significant increased levels of *OsIRO3 *transcripts, designated as OE-1 and OE-2.

### Plant cultivation conditions

Wild-type and transgenic plants were germinated and grown in culture solution prepared as described by Yoshida et al [36] except using EDTA-Fe instead of Citrate-Fe. The solution contained 1.425 mM NH_4 _NO_3 _, 0.323 mM NaH_2 _PO_4 _, 0.513 mM K_2 _SO_4 _, 0.998 mM CaCl_2 _, 1.643 mM MgSO_4 _, 0.009 mM MnCl_2 _, 0.075 μM (NH_4 _)_6 _Mo_7 _O_24 _, 0.019 mM H_3 _BO_3 _, 0.155 μM CuSO_4 _, 0.125 mM FeEDTA and 0.152 μM ZnSO4.

Rice seeds were germinated in distilled water for 2 days. After germination, 25 seedlings were transferred to a plastic net floating on the nutrient solution described above. For metal deficient treatments, two normally growth rice seedlings were transferred to K-, Fe-, Zn-, Cu- and Mn-free solution. Solution culture was changed every two days. After ten days of metal depletion treatments, root and leaf samples were harvested for RNA extraction and gene expression analysis. For Fe-deficient time-course analysis, rice seedlings were transferred to +Fe and -Fe solutions, root and shoot samples for RNA extraction were taken after 1, 3, 6 and 8 days,

For different Fe concentration treatments, three week old transgenic and wild-type rice plants were transferred to solution culture containing with 100, 10 or 1 μM EDTA-Fe. After 8 days of treatments, roots and leaves were sampled for RNA extraction, measurements of metal concentration, SPAD and shoot length measurements.

### Measurement of chlorophyll content

SPAD values (total chlorophyll content) were determined on the fully expanded youngest leaves of seedlings with a portable chlorophyll meter (SPAD-502; Minolta Sensing).

### Measurement of the Fe concentrations

To determine the concentrations of Fe in the rice plants, elemental analysis was conducted on seedlings grown under all treatments. Shoot and root samples were ground to a fine powder and digested with 5 mL of 11 M HNO_3 _for 5 h at 150°C. Metal concentrations were measured using Inductively Coupled Plasma Mass Spectrometry (ICP-MS, Agilent 7500ce, Santa Clara, CA, US).

### Quantitative RT-PCR

Total RNA was extracted from plant samples using TRIzol Reagent (Invitrogen, CA, USA) according to the manufacturer's recommendations. First-strand cDNAs were synthesized from total RNA using SuperScript II reverse transcriptase (Invitrogen, CA, USA). QRT-PCR was performed using SYBR Premix Ex Taq™ (Perfect Real Time) Kit (TaKaRa Biomedicals, Tokyo, Japan) on a LightCycler480 machine (Roche Diagnostics, Basel, Switzerland), according to the manufacturer's instructions. The amplification program for SYBR Green I was performed at 94°C for 10 s, 58°C for 10 s, and 72°C for 10 s. Triplicate quantitative assays were performed on each cDNA sample. The housekeeping gene *Actin *was used as an internal control. The relative level of expression was calculated using the formula 2-Δ(ΔCp). All the primers that were used for the QRT-PCR are given in Additional file 6.

### Phylogenetic analysis

Alignment and phylogenetic tree were conducted using default settings and the neighbor-joining algorithm of the MEGA version 4 [37] with 1000 bootstrap trials. Amino acid sequences of the bHLH TFs used for the analysis are listed in Additional file 1. Amino acid sequence similarity analysis was conducted in a website tool found at: http://www.ebi.ac.uk/Tools/emboss/align/index.html.

### Statistical analysis of data

For comparisons of treatments in Figure 4, a two sample t-test assuming unequal variances was performed with all samples compared to wild-type grown in three different levels of Fe supply. Significance was defined as p ≤ = 0.05.

## Abbreviations

bHLH: basic helix loop helix; FRO: ferric reductase/oxidase; IRT: iron regulated transporter; NA: nicotianamine; PS: phytosiderophore; YS: yellow stripe

## Authors' contributions

LZ, YY and LW did the physiological, gene expression analysis and transgenic work. LW and FW did the GFP fusion protein and subcellular localization work. LZ and HS participated in the design and coordination of the study. LZ, JW and HS wrote the manuscript. All authors read and approved the final manuscript.

## Supplementary Material

Additional file 1 **The amino acid sequences of bHLH transcription factors used for sequence similarity analysis and phylogenetic tree construction in Figure **3. Click here for file

Additional file 2 **Growth performance of the *OsIRO3 *over-expression lines and wildtype plant**. Click here for file

Additional file 3 ***OsIRO2 *cis element "G-box plus G" in the promoter region of *OsIRO3***. Click here for file

Additional file 4 **Expression of *OsIRO3 *(A) and *IDEF1 *(B) in different tissues and treatments**. Click here for file

Additional file 5 **Constructs of *OsIRO3 *over-expression binary vector**. Click here for file

Additional file 6 **Primer sequences used in QRT-PCR analysis**. Click here for file
