# Supplementary figures and images for: Identification of a novel iron regulated basic helix-loop-helix protein involved in Fe homeostasis in Oryza sativa
Source: BMC Plant Biol. 2010 Aug 11;10:166. doi: 10.1186/1471-2229-10-166 (PMC3017827; doi:10.1186/1471-2229-10-166)

## Slide 1
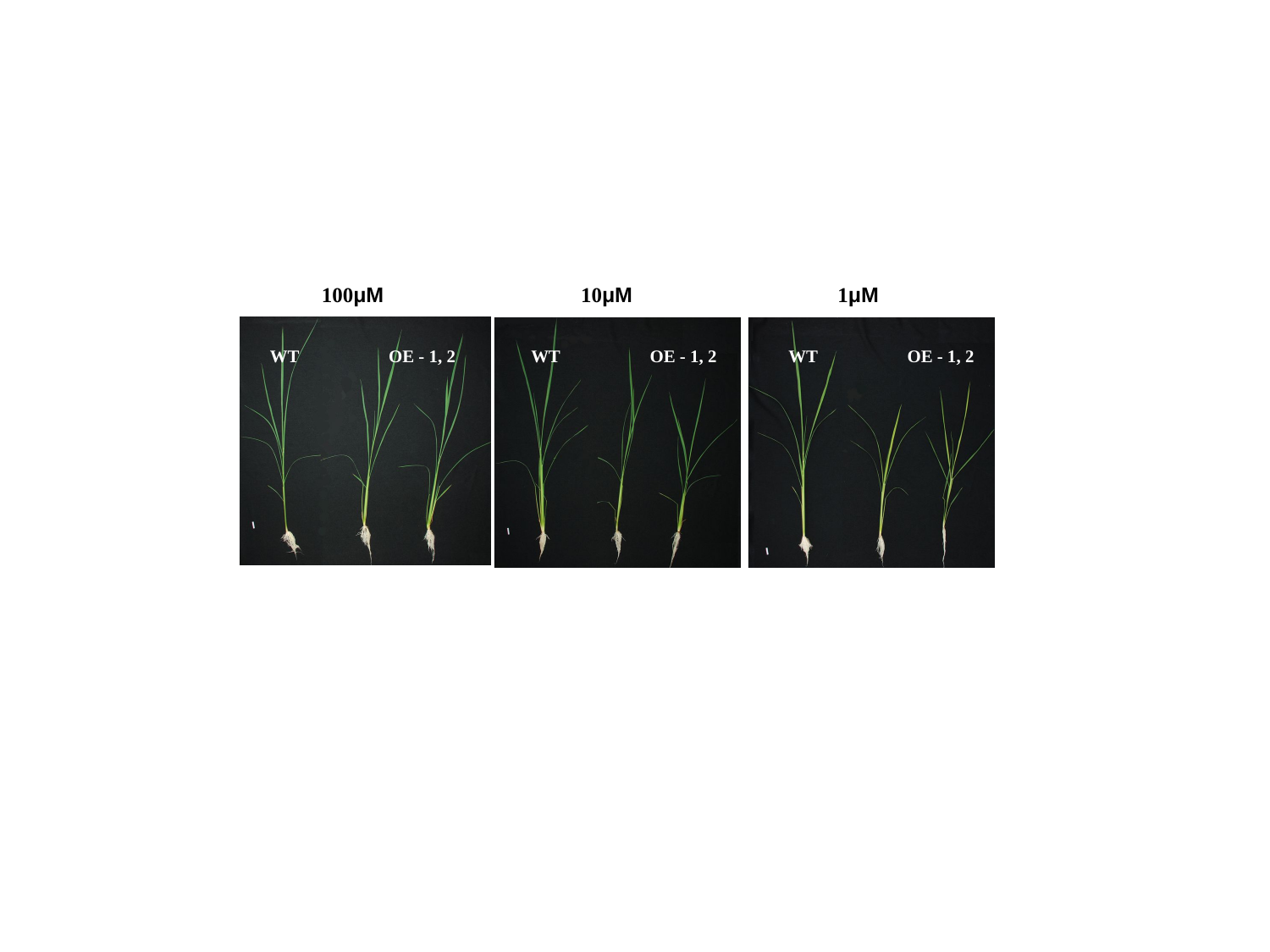

100μM
10μM
1μM
WT
OE - 1, 2
WT
OE - 1, 2
WT
OE - 1, 2

Supplement: Additional file 2 — Growth performance of the OsIRO3 over-expression lines and wildtype plant. [file 1471-2229-10-166-S2.PPT]

## Slide 1
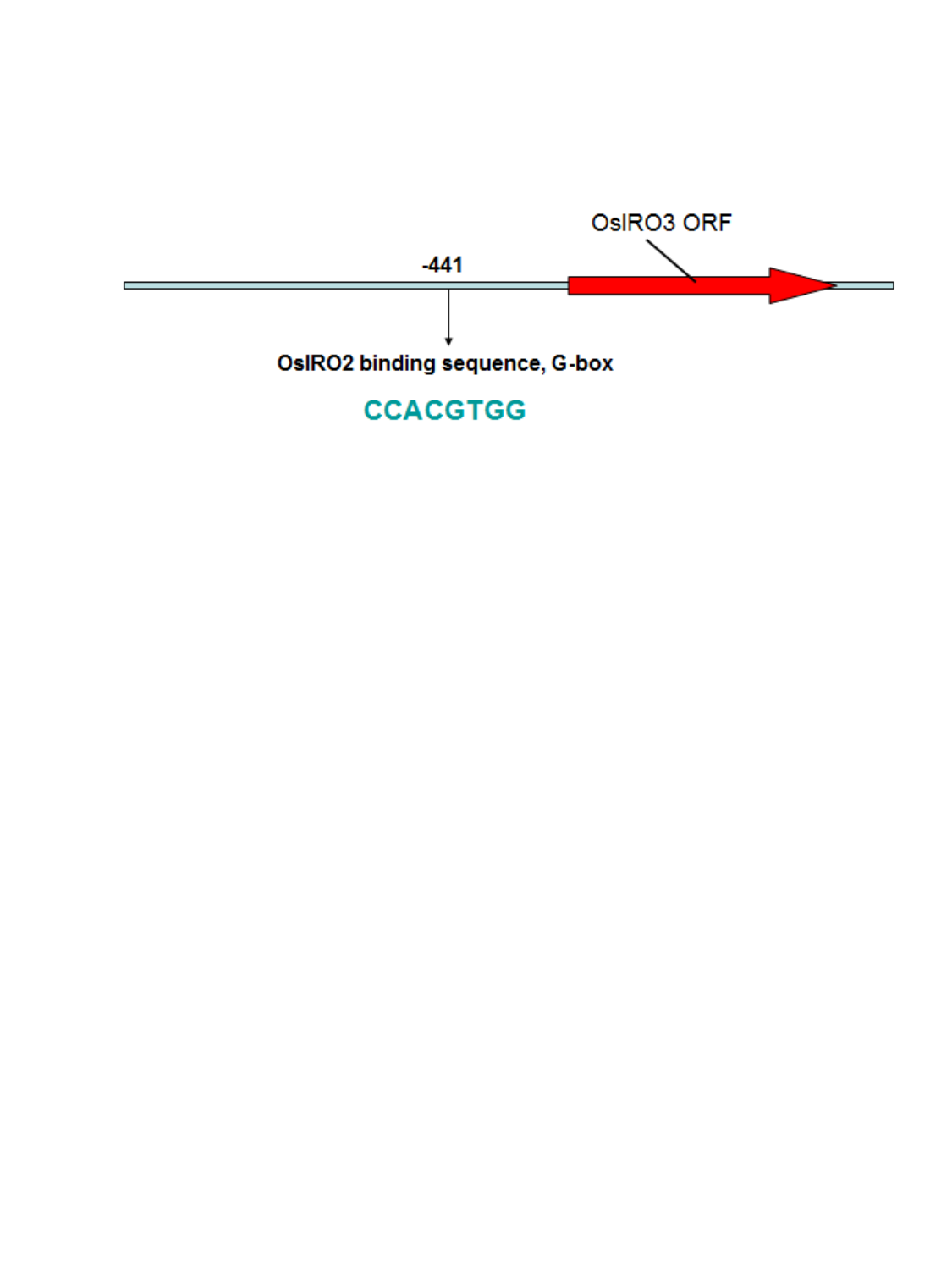

Supplement: Additional file 3 — OsIRO2 cis element "G-box plus G" in the promoter region of OsIRO3. [file 1471-2229-10-166-S3.PPT]

## Slide 1
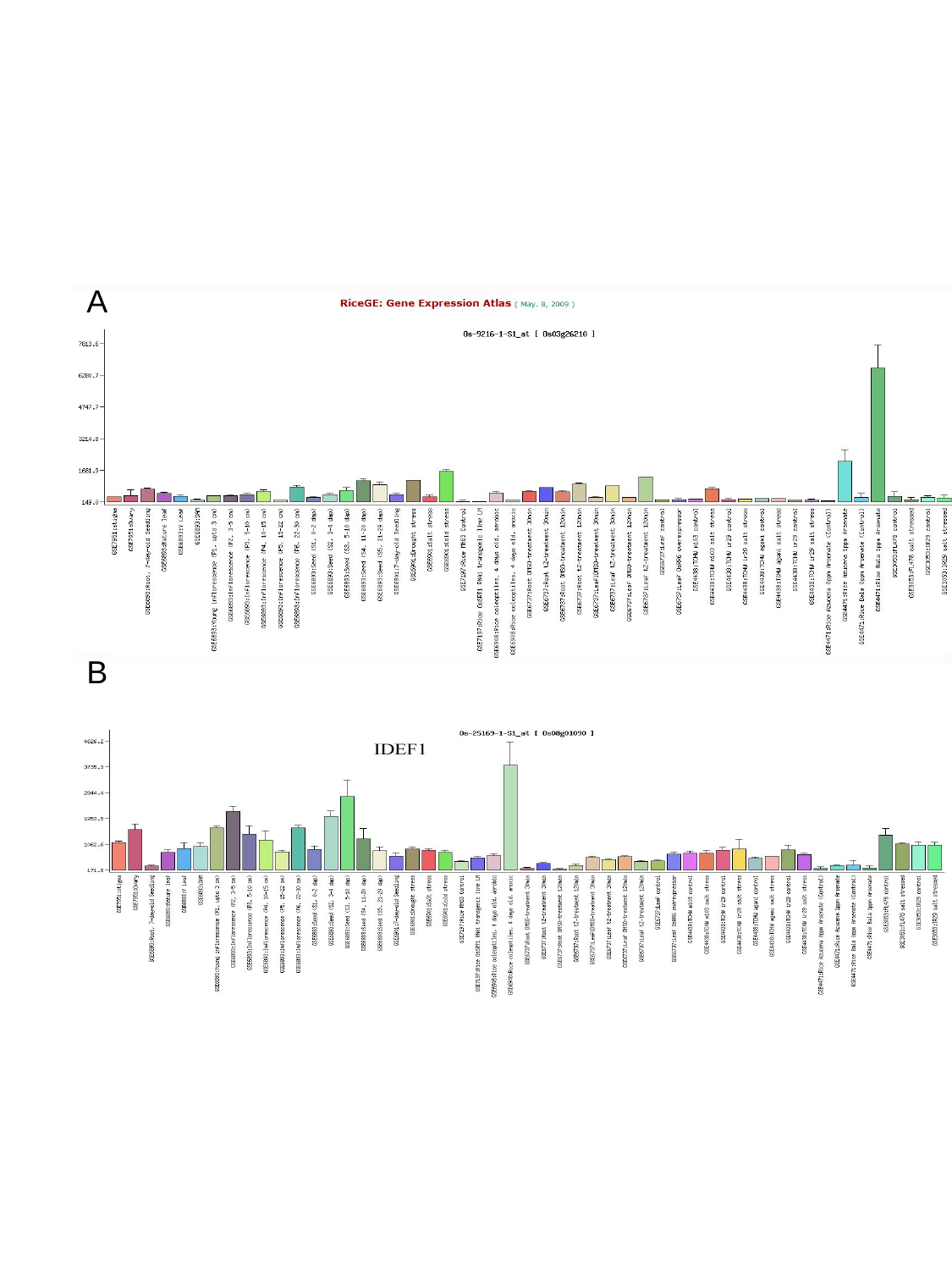

A
B

Supplement: Additional file 4 — Expression of OsIRO3 (A) and IDEF1 (B) in different tissues and treatments. [file 1471-2229-10-166-S4.PPT]

## Slide 1
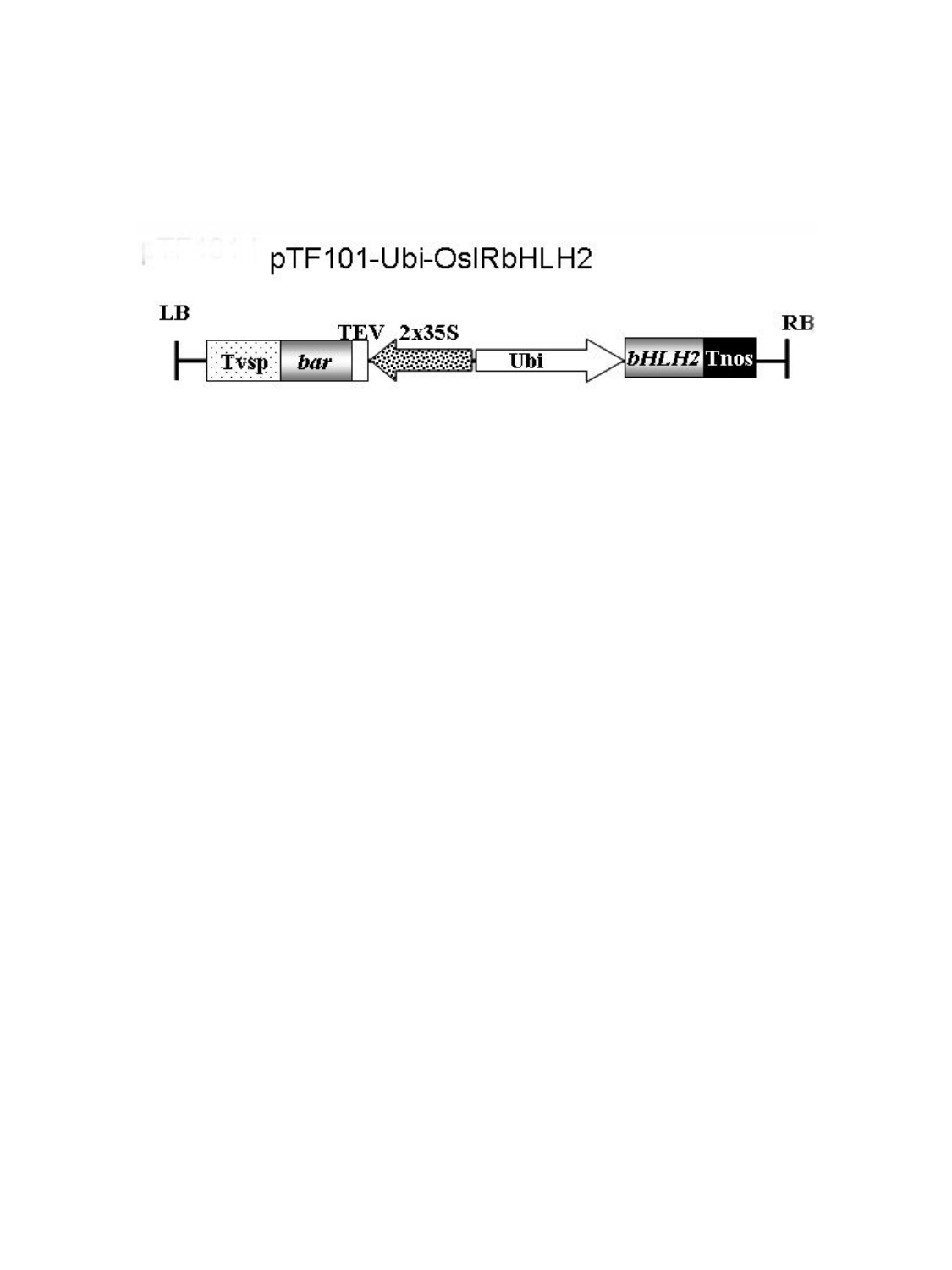

Supplement: Additional file 5 — Constructs of OsIRO3 over-expression binary vector. [file 1471-2229-10-166-S5.PPT]
