# Supplementary material for: Identification of a novel iron regulated basic helix-loop-helix protein involved in Fe homeostasis in Oryza sativa
Source: BMC Plant Biol. 2010 Aug 11;10:166. doi: 10.1186/1471-2229-10-166 (PMC3017827; doi:10.1186/1471-2229-10-166)
Supplement: Additional file 6 — Primer sequences used in QRT-PCR analysis. [file 1471-2229-10-166-S6.DOC]

**Gene** **Forward (5'-3') Reverse(5'-3')**

OsIRO3 GCGAGCTGGGTAATATGCTAGA ATCCGGGTGGTGTCAGTTAG

OsIRO2 GAAGGTCTTCACTTCATCAGTTCA TGATCGTTCCTTCACTTCTCTG

IDEF1 GTCTTCAGGCTGGGGATGT GGGATTTGTTGTCTGCTGATG
OsNAS1 CGGTTGAGAAGGCAGAAGAG TCGTCCGGCTGTTAGACG
OsNAS2 CGTCTGAGTGCGTGCATAGT CACAAACACAAACCGATACCA
OsIRT1 AGGTCGGTGCTCGTCTTCT TGTCCCTGTACACCCTGGTC
OsYSL15 GAGCTTCGCCATCGACAT TTGTTCATCTTGTTCCAAGCA
OsNRAMP1 GCAGCGACCTTACATTGGAC ACCGTACACTGTCGCACTTG
OsActin TCAGCAACTGGGATGATATGGAG GCCGTTGTGGTGAATGAGTAAC
